# Clostridium Difficile and Noncirrhotic Hyperammonnemia in a Patient With COVID-19 Infection

**DOI:** 10.7759/cureus.14533

**Published:** 2021-04-17

**Authors:** Sathishkumar Ramalingam, Kulothungan Gunasekaran, Harkesh Arora, Maheswari Muruganandam, Priyesh Padmanabhan

**Affiliations:** 1 Hospital Medicine, Lovelace Medical Center, Albuquerque, USA; 2 Pulmonary Critical Care, Yale New Haven Health at Bridgeport Hospital, Bridgeport, USA; 3 Rheumatology, University of New Mexico, Albuquerque, USA; 4 Cardiology, UnityPoint Health, Des Moines, USA

**Keywords:** covid-19, noncirrhotic hyperammonnemia, c. difficile, cdi, hyperammonemia

## Abstract

*Clostridium difficile* is a bacterial infection that usually presents with diarrhea and is mostly associated with previous antibiotics use. Patients with coronavirus disease 2019 (COVID-19) generally have respiratory symptoms but can also present with diarrhea. Noncirrhotic hyperammonemia is an infrequent presentation and is treated with lactulose. We report the case of a 40-year-old male who was admitted to our hospital with abdominal pain, diarrhea, shortness of breath, and confusion. During hospitalization, the patient tested positive for COVID-19 and *C. difficile*, and oral vancomycin was administered. His kidney functions improved, but he remained confused. His ammonia levels were elevated, and he was not treated with lactulose due to ongoing diarrhea secondary to *C. difficile* infection.

## Introduction

Patients with coronavirus disease 2019 (COVID-19) infection usually present with respiratory symptoms but can also present with diarrhea, coagulation abnormalities, and/or neurological symptoms [1-3]. The incidence of *Clostridium difficile* has reduced due to universal precaution and increased hand hygiene [4]. The use of antibiotics in COVID-19 pneumonia increases the risk of *C. difficile* infection (CDI), and noncirrhotic hyperammonemia in COVID-19 patients can cause altered mental status. Here, we present the case of a 40-year-old male with COVID-19 infection, CDI, and noncirrhotic hyperammonemia.

## Case presentation

**Figure 1:**
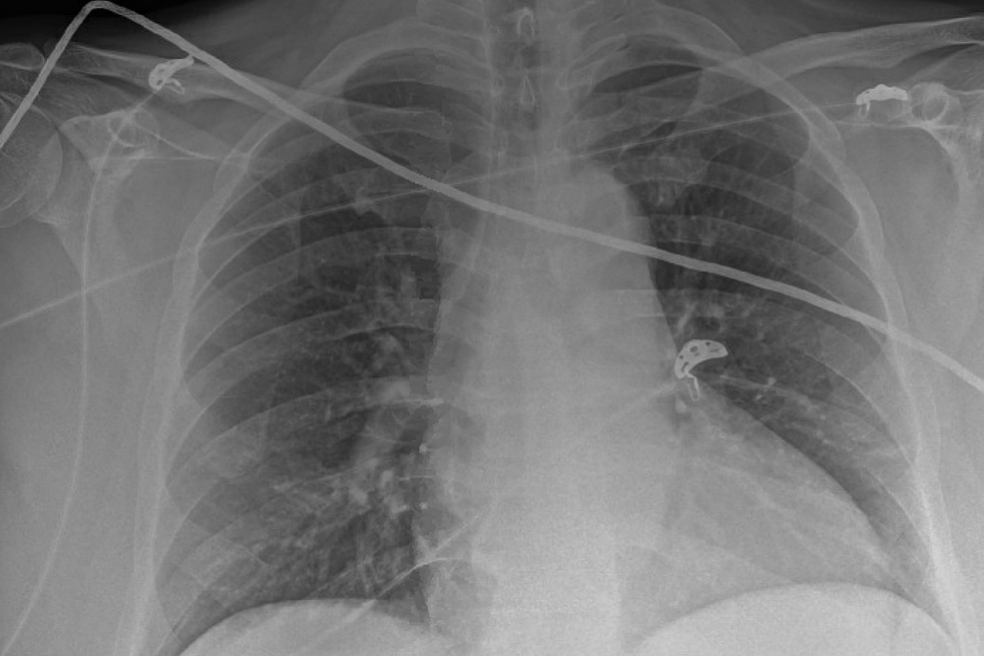
Chest X-ray showing increased bilateral interstitial marking

**Figure 2:**
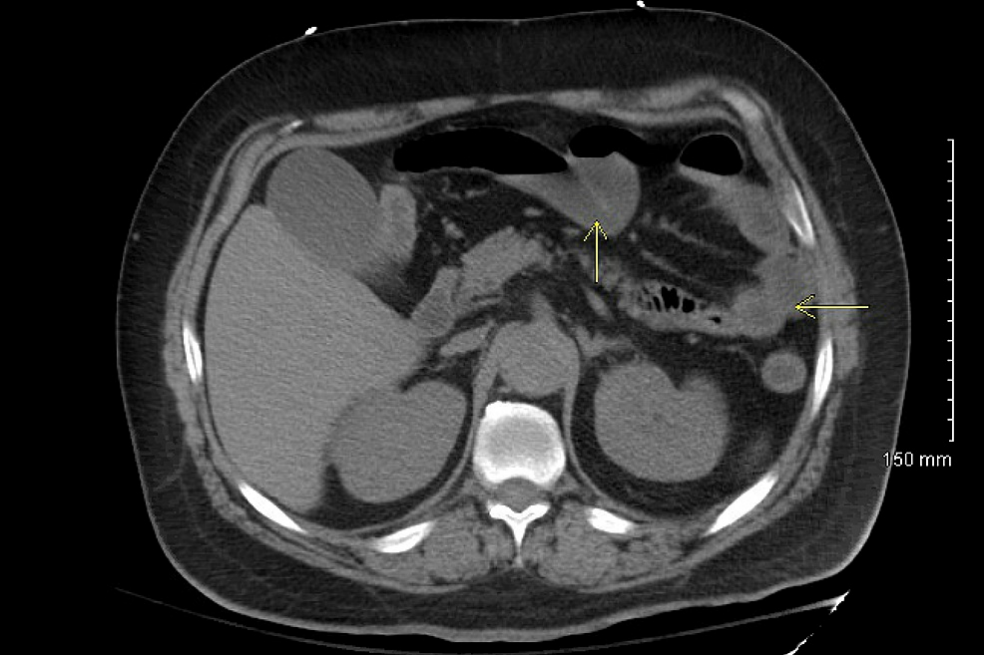
Computed tomography (CT) scan of the abdomen without contrast showing mild nonspecific fluid distension of small bowel and portions of colon consistent with enterocolitis

A 40-year-old non-alcoholic male with a past medical history of hypertension and type 2 diabetes mellitus was admitted with abdominal pain, diarrhea, shortness of breath, and confusion. His temperature was 98.7°F, heart rate was 78 bpm, oxygen saturation was 90% in room air, and blood pressure was 96/54 mmHg. On examination, abdominal tenderness was present, with reduced bilateral air entry and altered mental status. Laboratory studies showed elevated white blood cells (13.3 K/UL), and a CO_2_ level of 11 mmol/L, blood urea nitrogen (BUN) of 131 mg/dL, creatinine of 6.90 mg/dL, total bilirubin of 1.2 mg/dL, alanine aminotransferase (ALT) of 34 U/L, aspartate aminotransferase (AST) of 29 U/L, glucose of 131 mg/dL, lactic acid of 3.6 mmol/L, and creatine kinase level of 2,282 U/L. His oxygen requirement increased on day 2, requiring 3 L of oxygen. Chest X-ray showed increased bilateral interstitial marking (Figure 1). He continued to have increased abdominal pain and underwent a computed tomography (CT) scan of the abdomen without contrast, which showed fluid distension in the small bowel and colon consistent with enterocolitis (Figure 2). Polymerase chain reaction (PCR) for COVID-19 and PCR and enzyme immunoassay (EIA) *C. difficile* was positive, as he had persistent diarrhea. Remdesivir and steroids were not administered due to acute kidney injury and CDI; instead, intravenous fluids and oral vancomycin were administered. His kidney functions improved with intravenous fluids as recommended by the nephrology team. His renal functions improved on the fourth day of admission: creatinine was 2.10 mg/dL and BUN 69 was mg/dL. He remained confused; a CT scan of the head did not show any acute intracranial abnormalities, and he had an ammonia level of 102 umol/L. Lactulose was not started due to ongoing diarrhea secondary to CDI. His mentation improved, and his ammonia levels were reduced to 37 umol/L, and he was discharged on day 12.

## Discussion

COVID-19 presents mostly with respiratory symptoms, but other symptoms include diarrhea, fever, stroke, and coagulopathic abnormalities including deep vein thrombosis and pulmonary embolism. Universal precaution and hand hygiene have resulted in a reduction in CDI in hospitalized patients. Antibiotics are frequently used for the treatment of COVID-19 pneumonia and can increase the risk of CDI [5]. Antibiotic stewardship is vital in reducing the use of antibiotics in COVID-19 pneumonia. There must be high suspicion for CDI in COVID-19 patients with persistent diarrhea. The use of steroids in patients with COVID-19 and severe CDI has not been well studied and may complicate the management plan with acute respiratory failure. Our patient was started on fidaxomicin as he did not respond well to oral vancomycin. The optimal medical management of CDI in COVID-19 patients requires further study. Our patient was not on any antibiotics previously, and such patients require close monitoring because respiratory distress may mask life-threatening complications such as toxic megacolon.

Hyperammonemia refers to an increased ammonia level in the blood, and the most common cause is hepatic dysfunction. Hyperammonemia can occur in inborn errors of metabolism, such as carnitine deficiency, and urea cycle disorders, such as Ornithine transcarbamylase (OTC) deficiency. Medications such as valproic acid and carbamazepine can disrupt the urea cycle and can cause elevated ammonia levels. Hyperammonemia can also occur in seizures, starvation, and trauma, where there is increased muscle catabolism. Total parenteral nutrition, malignant myeloma, and infections caused by urease-producing bacteria such as Proteus *mirabilis*, *Escherichia coli*, and *Klebsiella* can cause hyperammonemia. Acute kidney injury can cause hyperammonemia as normal functioning kidneys excrete 20% of the body’s ammonia [6,7]. Honore et al. recently described elevated ammonia levels with normal liver functions in COVID-19 patients. Our patient’s altered mental status persisted even after the kidney functions improved, which may have been due to hyperammonemia secondary to COVID-19 [8]. The exact mechanism of elevated ammonia levels in COVID-19 patients is not yet understood. Lactulose is used to treat noncirrhotic hyperammonemia but was not started in our patient due to *C. difficile*, and his mentation and ammonia levels got better after his kidney functions and respiratory status improved.

## Conclusions

Patients with COVID-19 can present with diarrhea and must have a high index of suspicion for CDI. Antibiotic stewardship plays an essential role in reducing the use of antibiotics in COVID-19 patients and can reduce the occurrence of *C. difficile*. Our case presents a unique challenge of administering steroids to patients with CDI and COVID-19 infection. Noncirrhotic hyperammonemia should be considered in COVID-19 patients with altered mental status.
